# A Practical Cheoplastic Plate

**Published:** 1892-08

**Authors:** C. W. Staples

**Affiliations:** Lyndon, VT.


ARTICLE II.

### A PRACTICAL CHEOPLASTIC PLATE.

BY C. W. STAPLES, D. D. S., LYNDON, VT.

No arguments are needed to convince you of the superiority of metal over rubber and celluloid as a base for artificial plate, and it is equally an established fact that tin has a beneficial influence upon mucous membrane as well as upon dentine.

That there has been some reason why each successive alloy of tin with methods of working has not been practiced in any considerable proportion of cases, has crowded such bases out of the position that they should occupy.

It is my purpose to present a method or rather a combination of methods (for which I claim no originality), by which a lower plate, either partial or full, can be constructed in about the same time as that required for rubber, and overcome some of the former objection to such cheoplastic plate and still retain the advantages of metal.

Such a plate must be made so as to accommodate gum teeth as well as plain without adding any extra risk of breaking. In the ordinary methods the full lower cases have been too heavy and the gums cracked, either during construction or in after years by the softness of the metal allowing them to move slightly; this we overcome by using rubber attachment. Then in the case of lower partials, the patient was sure to bend and break the plate unless it was made thick and bungling, this we overcome by a wire spring of Dr. W. H. Dorrance's invention.

*Explanation.*—Proceed as usual with impression, model may be poured of plaster, but plaster with asbestos or whiting is safe. To this method fit accurately a piece of piano-wire, No. 14, 16 standard gauge along the arch so as to leave the arch about opposite the first molar; after fitting the wire to the arch bend each end inward at right angles with body of wire, then about ¼ inch from first bend make a second by bending wires upward forming an obtuse angle. This is done so that the wire will be held firmly in the plaster of the upper half of the flask; this done take a file and make a notch on each side of wire in the first bend of each end, this is done so that the wire will break in the proper place and easily when wanted; sandpaper the wire to remove all dirt from surface and dip first into muriate of zinc, then into melted tin, this is done so that the metals used for plate will flow along and become attached to the wire; the wire prepared, cover the model to just the extent that you wish the plate to cover the ridge when done. It is now necessary to decide whether to use a solid plate of metal or a rubber attachment. In nearly all cases if full lower, and if partials with much absorption, I use the latter, and have selected such a partial for description.

Warm the tinned wire and press into place, and cover the scar with a fresh piece of wax, which is now ready to flask. For this I find the Watt's flask most convenient, although the Weston is good and the one used in this case. In flasking, care must be used to have sufficient plaster under the ends of the wire to hold them firm and without breaking in the upper part of flask. With sharp knife make a groove around the edge of plate in upper part of flask just where edge of rubber will finish to, and is done so as to furnish a more secure attachment for rubber and a larger surface of metal next to the mouth.

For a more secure attachment, especially in full cases, I make several pits about ½ inch deep in upper part of flask over the ridge, these can be made with an old excavator sharpened like a screw-driver. Now cut a gate from each angle, this I make ample as it can do no harm and proves a great convenience.

The two halves of flask are now dried separately in a temperature that will not calcine the plaster; the oven of ordinary heating stove (as Stewart, Mogie, etc.) is a convenient place. When a mirror held over the warm flask will not gather the slightest moisture they will do to pour and not until then.

When dry the surface of the model should be rubbed with a piece of base plate wax to smoothen the surface and also to act as a flux for metal. The mould should be warm and the metal but very little above melting point when pouring, and should be cooled slowly to obtain a smooth casting. After separating the wire should be broken off with the finger. It will break just below the surface if the notches have been made as described. The small hole at each place where wire is broken is to be filled with some metal as that of which the plate is made, with a soldering copper (not tinned). To do this moisten the surface of plate about the hole with H Cl, or chloride of zinc, and place a piece of the metal over it and melt into place with warm copper. Now with a file smooth off plate in a rough form and fit to mouth; after fitting take the antagonism using plate just made as base plate, then proceed as usual with rubber attachment. Should you wish to make a solid plate after fitting the wire, you would proceed as usual with cheoplastic plates, excepting that after the case is on the articulator, the wire is to be put in place before the teeth are ground.

Any of the alloys of tin in use may be used for construction of this plate. While I have tried them all I like 15 parts silver to 85 of tin, although the addition of 3 per cent. of bismuth makes a good plate.—*Ohio Journal of Dental Science.*

